# Supplementary material for: Population Genetics and Anastomosis Group’s Geographical Distribution of Rhizoctonia solani Associated with Soybean
Source: Genes (Basel). 2022 Dec 19;13(12):2417. doi: 10.3390/genes13122417 (PMC9777564; doi:10.3390/genes13122417)
Supplement: Supplementary file 1 [file genes-13-02417-s001.zip › genes-2089318-supplementary.pdf]

Supplementary Tables

Table S1. GenBank accession numbers of DNA sequences from the rDNA ITS1-5.8S-ITS2 region of *R. solani* AG were used to determine AG's phylogenetic relationships and genetic diversity associated with soybean.

S. no.	AG/AG subgroup	Accession no.	Isolate	Pathogenicity	Geographic origin	Reference
1	AG1-IA	AF354060	1Rs	Foliar blight	USA	[37]
2	AG5	AF354078	10Rs	Damping off	Japan	"
3	AG5	KX118386	USA_AG-5	Damping-off, root and hypocotyl rot	USA	[5]
4	AG3	KX118385	USA_AG-3	"	USA	"
5	AG7	KX118384	ST81548	"	USA	"
6	AG11	KX118382	SP_19b	"	USA	"
7	AG11	KX118381	SP_19a	"	USA	"
8	AG5	KX118377	Rh0911029	"	USA	"
9	AG2-1	KX118376	Rh051324	"	USA	"
10	AG2-1	KX118375	Rh051307	"	USA	"
11	AG2-2IIIB	KX118373	PDONS_13_8_1	"	Canada	"
12	AG2-2IIIB	KX118372	ONS02_18	"	Canada	"
13	AG11	KX118367	K_4_18b	"	USA	"
14	AG2-1	KX118363	KARS02_5_1	"	USA	"
15	AG7	KX118362	KARS02_2_5	"	USA	"
16	AG7	KX118361	KARS02_1_9	"	USA	"
17	AG7	KX118360	KARS02_1_8	"	USA	"
18	AG7	KX118359	KARS02_1_6	"	USA	"
19	AG11	KX118358	KARS02_1_20	"	USA	"
20	AG11	KX118356	KARS02_1_11	"	USA	"
21	AG11	KX118355	HPIN22A	"	USA	"
22	AG7	KX118354	EV_7	"	USA	"
23	AG4-HGI	KX118353	EV_6	"	USA	"
24	AG4-HGI	KX118352	EV_3	"	USA	"
25	AG7	KX118351	EV_19	"	USA	"
26	AG2-2IIIB	KX118350	ER_4	"	USA	"
27	AG2-2IIIB	KX118349	ER_2	"	USA	"
28	AG2-2IIIB	KX118348	ER_19b	"	USA	"
29	AG2-2IIIB	KX118347	ER_19a	"	USA	"
30	AG2-2IIIB	KX118346	ER_15	"	USA	"
31	AG2-2IIIB	KX118345	DK_8	"	USA	"

32	AG2-2IIIB	KX118344	DK_11	"	USA	"
33	AG3	KX118343	Cfar_500_6	"	USA	"
34	AG3	KX118342	Cfar_500_3	"	USA	"
35	AG3	KX118341	Cfar_500_10a	"	USA	"
36	AG3	KX118340	Cfar_500_1	"	USA	"
37	AG4-HGIII	KX118339	BVT_3	"	USA	"
38	AG4-HGIII	KX118338	BVT_28	"	USA	"
39	AG7	KX118337	BVT_20	"	USA	"
40	AG11	KX118336	BVT_18	"	USA	"
41	AG7	KX118335	BVT_16	"	USA	"
42	AG2-2IIIB	KX118334	65L-2	"	USA	"
43	AG4-HGIII	KX118331	AG-4_Carling	"	USA	"
44	AG4-HGIII	KX118330	AG-4_Nelson	"	USA	"
45	AG4-HGIII	KX118329	Rh051339	"	USA	"
46	AG2-2	KX118328	AG2-2_Nelson	"	USA	"
47	AG2-2-IIIB	KX118397	X42210_b	"	USA	"
48	AG3	KX118396	X248_3bKH	"	USA	"
49	AG11	KX118394	X12SDSa	"	USA	"
50	AG2-2IIIB	KX118392	X12Rs41	"	USA	"
51	AG2-2IIIB	KX118391	WONS_13_8_5	"	USA	"
52	AG2-2IIIB	KX118390	WONS_13_12_3	"	USA	"
53	AG-1	JF701709	RAPS3	Web/foliar blight/wet rot	India	[68]
54	AG2-3	FJ435110	CSL1934	Damping off	Japan	[44]
55	AG2-3	FJ435099	CSL1866	Damping off	Japan	"
56	AG7	AF153793	Roth32	none	USA	[77]
57	AG11	AF354115	Roth24		USA	[78]
58	AG11	AF354114	Roth16		USA	"
59	AG5	AF354112	19Rs		Japan	"
60	AG4	AF354081	BN38		USA	"
61	AG1-IA	DQ173075	SJ142-2	Leaf blight	Brazil	[33]
62	AG1-IA	DQ173074	SJ140	"	Brazil	"
63	AG1-IA	DQ173073	SJ134	"	Brazil	"
64	AG1-IA	DQ173072	SJ133	"	Brazil	"
65	AG1-IA	DQ173071	SJ129	"	Brazil	"
66	AG1-IA	DQ173070	SJ127	"	Brazil	"
67	AG1-IA	DQ173069	SJ121	"	Brazil	"

68	AG1-IA	DQ173068	SJ093	"	Brazil	"
69	AG1-IA	DQ173067	SJ080	"	Brazil	"
70	AG1-IA	DQ173066	SJ064	"	Brazil	"
71	AG1-IA	DQ173065	SJ053	"	Brazil	"
72	AG1-IA	DQ173064	SJ048	"	Brazil	"
73	AG1-IA	DQ173063	SJ047	"	Brazil	"
74	AG1-IA	DQ173062	SJ044	"	Brazil	"
75	AG1-IA	DQ173061	SJ040	"	Brazil	"
76	AG1-IA	DQ173060	SJ036	"	Brazil	"
77	AG1-IA	DQ173059	SJ031-6	"	Brazil	"
78	AG1-IA	DQ173058	SJ031-5	"	Brazil	"
79	AG1-IA	DQ173057	SJ023	"	Brazil	"
80	AG1-IA	DQ173056	SJ021	"	Brazil	"
81	AG1-IA	DQ173055	SJ015	"	Brazil	"
82	AG1-IA	DQ173054	SJ014-2	"	Brazil	"
83	AG1-IA	DQ173053	SJ013	"	Brazil	"
84	AG1-IC	DQ173077	AG-1 IC	"	USA	"
85	AG1-IB	DQ173076	AG-1IB	"	USA	"
86	AG1-IA	DQ173052	Rhs-9F1	"	Brazil	"
87	AG1-IA	DQ173051	Rhs-4F1	"	Brazil	"
88	AG1-IA	DQ173050	Rhs-3F6	"	Brazil	"
89	AG1-IA	DQ173049	Rhs-3F1	"	Brazil	"
90	AG1-IA	DQ173048	AG1IArJ	"	Japan	"
91	AG1-IA	DQ173047	AG1IAmJ	"	Japan	"
92	AG4-HGII	AY270005	Not available	Hypocotyl rot	Brazil	[32]
93	AG2-2IIIB	AY270015	SJ07		Brazil	[78]
94	AG1-IA	AY270013	SJ19	Foliar blight	Brazil	[32]
95	AG1-IA	AY270012	SJ57	Foliar blight	Brazil	"
96	AG1-IA	AY270011	SJ67	Foliar blight	Brazil	"
97	AG1-IA	AY270010	SJ16	Foliar blight	Brazil	"
98	AG1-IA	AY270009	SJ26	Foliar blight	Brazil	"

99	AG1-IA	AY270008	SJ24	Foliar blight	Brazil	“
100	AG1-IA	AY270007	SJ34	Foliar blight	Brazil	“
101	AG1-IA	AY270006	SJ28	Foliar blight	Brazil	“
102	AG4-HGII	AY270004	SJ03	Hypocotyle rot	Brazil	“
103	AG4-HGII	AY270003	SJ02	Hypocotyle rot	Brazil	“
104	AG4-HGII	AY270002	SJ05	Hypocotyle rot	Brazil	“
105	AG4-HGII	AY270001	SJ01	Hypocotyle rot	Brazil	“
106	AG-7	MW410857	Unknown	Damping off	Taiwan	[42]
107	AG-7	MW410858	Unknown	Damping off	Taiwan	[42]
108	AG-1 IF	MK734418	Unknown	Foliar blight	Brazil	[64]
109	AG-9	KX118333	AG-9_Carling	Unknown	USA	[5]
110	AG-6	KX118332	AG-6_Carling	Unknown	USA	[5]
111	AG-1-IB	KY172991	ppj-3	Unknown	India	Unpublished
112	AG-1-IB	JQ911746	AG_1-IB	Unknown	India	Unpublished
113	AG-2-3	U57742	23R03	Unknown	Japan	Unpublished
114	AG-2-3	U57741	23R02	Unknown	Japan	Unpublished
115	AG-2-3	U57740	23R01	Unknown	Japan	Unpublished

Table S2. The best model used for ML and NJ analysis in this study

Model	#Param	BIC	AICc	lnL	Invariant	Gamma	R	Freq A	Freq T	Freq C	Freq G	A=>T	A=>C
TN93+G	206	9224.6	7383.2	-3484.9	n/a	0.25	1.41	0.30	0.30	0.20	0.20	0.06	0.04
T92+G+I	207	9235.6	7385.3	-3484.9	0.01	0.25	1.41	0.30	0.30	0.20	0.20	0.06	0.04
HKY+G	208	9235.8	7376.7	-3479.6	n/a	0.25	1.41	0.26	0.33	0.21	0.20	0.07	0.04
TN93+G	209	9241.4	7373.3	-3476.9	n/a	0.25	1.42	0.26	0.33	0.21	0.20	0.07	0.04
HKY+G+I	209	9246.2	7378.1	-3479.3	0.01	0.25	1.41	0.26	0.33	0.21	0.20	0.07	0.04
T92+G	210	9252.3	7375.3	-3476.8	0.02	0.26	1.42	0.26	0.33	0.21	0.20	0.07	0.04
K2+G	205	9261.2	7428.9	-3508.7	n/a	0.26	1.37	0.25	0.25	0.25	0.25	0.05	0.05
GTR+G	212	9271.0	7376.1	-3475.2	n/a	0.25	1.43	0.26	0.33	0.21	0.20	0.06	0.04
K2+G+I	206	9272.2	7430.9	-3508.7	0.01	0.26	1.37	0.25	0.25	0.25	0.25	0.05	0.05
GTR+G+I	213	9281.6	7377.8	-3475.1	0.02	0.26	1.43	0.26	0.33	0.21	0.20	0.06	0.04
JC+G	204	9346.6	7523.1	-3556.8	n/a	0.26	0.50	0.25	0.25	0.25	0.25	0.08	0.08
JC+G+I	205	9357.5	7525.1	-3556.8	0.02	0.26	0.50	0.25	0.25	0.25	0.25	0.08	0.08
T92+I	206	9392.9	7551.6	-3569.0	0.47	n/a	1.33	0.30	0.30	0.20	0.20	0.06	0.04
HKY+I	208	9406.3	7547.1	-3564.8	0.47	n/a	1.33	0.26	0.33	0.21	0.20	0.07	0.04
TN93+I	209	9412.0	7543.8	-3562.1	0.47	n/a	1.33	0.26	0.33	0.21	0.20	0.07	0.04
K2+I	205	9420.7	7588.3	-3588.4	0.47	n/a	1.33	0.25	0.25	0.25	0.25	0.05	0.05
GTR+I	212	9441.3	7546.4	-3560.4	0.47	n/a	1.33	0.26	0.33	0.21	0.20	0.07	0.04
JC+I	204	9504.2	7680.8	-3635.6	0.48	n/a	0.50	0.25	0.25	0.25	0.25	0.08	0.08
T92	205	9654.3	7822.0	-3705.2	n/a	n/a	1.21	0.30	0.30	0.20	0.20	0.07	0.05
HKY	207	9667.3	7817.1	-3700.8	n/a	n/a	1.22	0.26	0.33	0.21	0.20	0.07	0.05
K2	204	9669.5	7846.1	-3718.3	n/a	n/a	1.20	0.25	0.25	0.25	0.25	0.06	0.06
TN93	208	9670.3	7811.1	-3696.8	n/a	n/a	1.22	0.26	0.33	0.21	0.20	0.07	0.05
GTR	211	9683.0	7797.0	-3686.7	n/a	n/a	1.23	0.26	0.33	0.21	0.20	0.07	0.05
JC	203	9751.8	7937.2	-3764.9	n/a	n/a	0.50	0.25	0.25	0.25	0.25	0.08	0.08

Abbreviations: TR: General Time Reversible; HKY: Hasegawa-Kishino-Yano; TN93: Tamura-Nei; T92: Tamura 3-parameter; K2: Kimura 2-parameter; JC: Jukes-Cantor
